# Being the next of kin of an older person living in a nursing home: an interview study about quality of life

**DOI:** 10.1186/s12877-019-1343-4

**Published:** 2019-11-21

**Authors:** Helena Rosén, Lina Behm, Birgitta Wallerstedt, Gerd Ahlström

**Affiliations:** 1 Department of Health Sciences, Faculty of Medicine, Lund University, Lund, SE-221 00 Sweden; 2 Faculty of Health Science, Kristianstad University, Kristianstad, SE-291 88 Sweden; 3 Center for Collaborative Palliative Care, Department of Health and Caring Sciences, Faculty of Health and Life Sciences, Linnaeus University, SE-351 95 Växjö, Sweden

**Keywords:** Quality of life, Next of kin, Family member, Implementation, Older person, Nursing home, Palliative care, Qualitative content analysis, Relatives, Significant others

## Abstract

**Background:**

The length of stay in nursing homes before death in Sweden has significantly decreased, and nearly one-third of people die within 6 weeks of entering a nursing home. Support for the next of kin is one of the cornerstones of palliative care, but the principles are not always adhered to as recommended when caring for the elderly, which can affect the quality of life of their next of kin. The aim of this study was to explore the experiences of quality of life among the next of kin of older persons who live in nursing homes before an educational intervention of palliative care.

**Methods:**

This is an explorative qualitative interview study with 40 next of kin using qualitative content analysis performed at baseline before the implementation of the principles of palliative care in nursing homes.

**Results:**

The next of kin’s experiences of quality of life were expressed in three themes: Orientation to the new life situation, Challenges in their relationship and the Significance of the quality of care in the nursing home. The next of kin experienced a sense of relief, although the older person was constantly on their minds, and they could feel lonely. The difference in the couple’slife situations was experienced as burdensome by the next of kin. The challenges in the relationship were described as stressful, related to a guilty conscience and the older person’s vulnerability. The nursing home could be a context facilitating good relations. The perceptions of quality of care in terms of person-centredness affected the quality of life of the next of kin.

**Conclusions:**

The findings show that four factors are decisive for the quality of life of next of kin: the relationships within the family, the degree of relief that nursing home care entails as compared to home care, the older person’s health status and whether the care is person-centred. Increased knowledge and education regarding palliative care in nursing homes are needed to better meet the needs of next of kin. Implementation of palliative care should take into account the need for support for next of kin.

**Trial registration:**

NCT02708498, 15 March 2016.

## Background

The end of life (EoL) often involves relocation for an older person and a transition not only for the older person but also for his or her next of kin [1] which might be stressful and challenging for both people. One common site at the EoL is the nursing home [2], where the length of stay before death in Sweden decreased by more than 10 times between 2006 and 2012 [3]. Nearly one-third of older people who move into a nursing home die within 6 weeks, and the two main causes of death among older people are circulatory diseases (42.2%) and dementia (22.7%) [4]. Being the next of kin to an older person in a nursing home has been described as inducing a guilty conscience and feelings of insufficiency in the next of kin [5] as well as a dependence on staff to maintain control over the situation [5]. Support for the next of kin in this situation is one of the cornerstones of palliative care [6, 7]. However, the principles of palliative care are not always practiced as recommended for the care of older persons [6] which might influence the next of kin’s quality of life (QoL).

The next of kin have been shown to continue their caregiving tasks even if formal care is available [8]. The next of kin describe this as a balancing act, with feelings of wanting to maintain control conflicting with feelings of wanting to relinquish responsibility for the older person’s care [9]. Being the next of kin of an older person at the EoL has been described as being closely involved in the transition from the point when the older person becomes ill until the EoL [10]. This involvement sometimes requires the next of kin to adjust to the older person’s situation, which affects their own life situation and results in social isolation [11], has an impact on their health and leads to difficulties in managing their ordinary daily lives (such as work) [10]. A Norwegian study of 15 next of kin and 20 older persons in ten nursing homes found that the next of kin had poor knowledge about the EoL [12]. Consistent with this finding, a systematic review of the next of kin in EoL care (EoLC) [13] showed that being the next of kin at the EoL was a new situation for many people. This situation put them in a vulnerable position. The same study [13] showed that the next of kin often experienced their situation at the EoL as burdensome and as imposing increased responsibility, which could have negative consequences. Furthermore, the ratings of physical health and QoL have been shown to be lower for the next of kin in palliative care than for those in curative care [14]. The next of kin have also reported low satisfaction with their contact with staff, their influence on care, and meaningful activities for their older relatives [15]. Despite these deficiencies, research concerning intervention to support the QoL among the next of kin of older persons in nursing homes is sparse.

The WHO (2002) defines QoL as individuals’ perceptions of their position in life in the context of the culture and value systems in which they live and in relation to their goals, expectations, standards, and concerns. In an interview study of Vellone, Piras, Talucci and Cohen [16], a good QoL for 32 informal caregivers of patients with Alzheimer’s disease involved experiences of serenity, tranquillity, psychological well-being, freedom, good health and a good financial status. Other aspects of importance for an improved QoL of the next of kin were that the patient had good health, that they felt independent from the patient and that they received more help with caregiving (17). Having to make EoL decisions for an older family member could both increase and ease the caregiver burden for the next of kin depending on the expectations of the older person, the family relationship and how much responsibility the next of kin wants to assume [17].

The Swedish National Board of Health and Welfare [18] has set the goal to provide everyone who needs it with access to palliative care based on the WHO guidelines for palliative care [19], regardless of diagnosis, care form, age or location in the country. The palliative care approach aims to improve QoL for persons and their next of kin who face problems associated with the EoL [20] which includes communication, relationships, and support for next of kin. As part of meeting this goal, the National Board of Health and Welfare (2013) developed a knowledge base for good palliative care [21]. This knowledge base aims to support healthcare providers in developing palliative care and ensuring quality. As previous experience shows that there is a large gap between developing documents and practice, the Implementation of Knowledge-Based Palliative Care in Nursing Homes (KUPA) project was developed and initiated to implement the knowledge base in everyday care. The KUPA project aimed to implement a palliative care approach in nursing homes through educational intervention with staff [22, 23]. Thus, the aim of this study was to explore the experiences of QoL among the next of kin of older persons who live in nursing homes before an educational intervention of palliative care.

## Methods

The design consisted of an explorative qualitative interview study with the next of kin using qualitative content analysis [24, 25]. The interviews were conducted at baseline before implementation of the principles of palliative care in nursing homes through an educational intervention with staff and front-line managers in the KUPA project, the aim of which was to achieve the best possible palliative care and QoL for the next of kin (16).

### Nursing homes in Sweden

Nursing homes in Sweden are tax-funded and are the responsibility of the municipal councils. Every citizen is granted equal access to health care, elder care, and social services based on each person’s need for support. The current Swedish policy for older people is “Aging in place”, which means enabling older people to continue to live in their own homes for as long as possible and providing assistance if they need health care due to illness and multi-morbidity [26]. The needs of older people who receive home care are assessed by social workers in the municipality, and when the need for care becomes too great, they assess and grant the right to an apartment in a nursing home. This normally occurs when a person becomes too sick or frail to manage everyday life at home. In nursing homes, residents have their own apartments, and 24-h care is provided. Nursing home staff consist of assistant nurses (the largest group), registered nurses, occupational therapists, and physiotherapists.

### Educational intervention and the KUPA project

A considerable hindrance to providing palliative care in nursing homes is the lack of knowledge and training among staff members regarding the principles of palliative care [27]. The KUPA project aimed at addressing this obstacle through an educational intervention in twenty nursing homes in two counties in southern Sweden. The intervention consisted of five seminars over 6 months for staff and front-line managers in 10 nursing homes, with 10 other nursing homes acting as controls [22]. The evaluation is conducted through a non-randomized experimental design with quantitative and qualitative methods and pre- and post-assessments [22, 28].

### Sampling and participants

The next of kin in this study were recruited from 20 nursing homes, for a total of 40 participants from the two counties [22]. The inclusion criteria were that they were the next of kin of an older person at one of the nursing homes included in the KUPA project, were able to speak and understand Swedish and visited the older person at the nursing home regularly. The next of kin were selected by a contact person (a nurse assistant, a manager or an administrative person) at each participating nursing home according to the inclusion criteria. The next of kin were informed about the study and then asked if they were interested in participating. If they agreed, the contact person then gave their name and contact information to the researchers. The researchers contacted the next of kin and provided oral information about the study and again asked if they were willing to participate. If the next of kin consented to participate, a time and place for the interview were agreed upon. Before the interview began, further oral and written information about the study was provided, and written consent was signed by the next of kin. The interviews were normally performed in a conference room at the nursing homes or, in some instances, at the private home of the next of kin. Forty next of kin participated in the study (see Table 1).

**Table 1:** Characteristics of the participating next of kin (*n* = 40)

	Number	Percent
Age, years		
40–49	1	2.5
50–59	11	27.5
60–69	18	45
70–79	8	20
80–89	2	5
Gender		
Men	10	25
Women	30	75
Marital status		
Married/living together	32	80
Unmarried/divorced	6	15
Widower/widow	2	5
Relation to the older person		
Husband/wife	7	17.5
Daughter/son	31	77.5
Sibling	1	2.5
Other	1	2.5
Educational level^a^		
Elementary school	9	25
High school	8	22
Trade school	4	11
University/college	15	42
Work status		
Full-time	13	32.5
Part-time	9	22.5
Not working	18	45
The frequency of visits to the older person		
Every day	6	15
Weekly	31	77.5
Monthly	2	5
Yearly	1	2.5

^a^4 missing

### Interviews

Four researchers, all of whom are registered nurses with experience working in nursing homes, conducted the interviews, and all the interviews were performed in a similar manner based on a semi-structured interview guide. The interview began with the following two open-ended questions: “How do you experience your situation of being a next of kin?” and “How would you describe your quality of life in the past week?” Follow-up questions were asked to increase the richness of the next of kin’s answers about their physical health, well-being/mental health, social well-being/social relationships, and satisfaction with their life situation. The number and formulation of the follow-up questions depended on how comprehensive the preliminary answers were. The follow-up questions could be expressed as “Could you tell me more about your. ….?”, “What did you think in that situation?” and “What did you do in that situation?” The interviews were conducted from April 2015 to May 2016 and lasted as long as 77 min (mean = 42 min). All of the interviews were recorded and then transcribed verbatim.

### Data analysis

The interviews were analysed via qualitative thematic content analysis, a method by which sub-themes and themes are identified through a systematic process of coding data based on interpretation of the content of a text [24, 25]. This method was applied in this study according to the following steps:

1. Initially, the first author (HR) listened to all of the recorded interviews, and the transcribed interviews were read through several times to obtain an overall impression of their content in respect of the experienced QoL.
2. During the second step, meaning units consisting of one or more sentences or paragraphs of a narrative were identified from the transcribed interview text.
3. In the third step, the meaning units were coded and assigned preliminary denominations, and this coding process consisted of the identification of the manifest and latent content of each meaning unit (text segment). The second and third steps were performed with NVIVO software, QSR International Pty Ltd. 651 Doncaster, Victoria, Australia.
4. In the fourth step, the co-author (GA) also read the meaning units several times to assign preliminary denominations. The entire body of interview text was used as the reference material. Independent manifest and latent analyses were conducted by the researcher (GA) on the basis of identified similarities and differences between the meaning units.
5. In the fifth step, both researchers (HR and GA) compared and discussed the analyses they performed separately in the previous steps in a series of meetings. The reviews of the similarities and differences between the codes and the preliminary denominations developed successively to themes and sub-themes.
6. In the final step, all of the authors reviewed the findings until a consensus was reached and the themes and sub-themes were established. A selection of representative quotations was made to illustrate the findings.

## Results

The next of kin described briefly at the beginning of the interview some general aspects of a high QoL in terms of good health and a good financial situation. They looked after their own health through exercise and a proper diet. When they were “in step with life” and were able to “handle all the pieces of the daily life puzzle”, they felt free and were pleased with their homes and their neighbors and close relationships.

The next of kin spoke about their QoL qua next of kin with particular reference to the older person’s situation and the care that this person received. The following three themes emerged from the analysis: (1) Orientation to the new life situation; (2) Challenges in the relationship; and (3) The significance of the quality of care in the nursing home. Each theme comprised 2–4 sub-themes (Table 2).

**Table 2:** Themes and sub-themes of quality of life among the next of kin of older persons who live in nursing homes (*n* = 40)

Themes	Sub-themes
Orientation to the new life situation	Sense of relief
	Constantly on one’s mind
	Feeling alone
	Couples’ different life situations
Challenges in the relationship	Enabling good relations
	Relationship marked by a guilty conscience
	Encountering vulnerability
	Strained relations
The significance of quality of care in the nursing home	Satisfaction and appreciation
	Lack of person-centredness

### Orientation to the new life situation

#### Sense of relief

The next of kin described what it was like for them when the older person was still living at home. They had to be constantly on hand to attend to the person’s needs; therefore, it was a great relief when the person moved into a nursing home. They experienced a sense of security in knowing that there were staff available 24 h a day, in contrast to what it was like when the care was provided by a home help service. The next of kin felt that the older person shared this sense of security. With this new-found sense of security, the next of kin felt satisfied with their QoL. It was a relief that the older person was in a nursing home (whatever reservations there might be about the quality of the care). They now had more time of their own.

*Things were very different before. I can tell you it was hard for me, it really was. But now it’s as if I’ve been set free. I think I’ve got a very good life. It gives me a sense of security knowing that Mum’s being looked after at the nursing home. Knowing that the staff are doing all they can to make things better for her makes things better for me as well,* much *better. I feel calm, safe and secure. I think my life’s good.* (Daughter)

Previously, the next of kin’s visits to the older person had been very much a question of providing practical help. Now, however, it was a joy to just be together.

*I mean, it’s so lovely now to go and see Dad and just sit and talk, have a cup of coffee or go out together, without feeling that there are a lot of things that need doing. No disrespect to the home help service.* (Daughter)

#### Constantly on one’s mind

The next of kin indicated that it was important that the older person have as good a life as possible. They strove to ensure that staff, other residents and visitors respected the older person’s integrity. The older person was constantly on the next of kin’s mind, which resulted in anxiety and a burden that restricted their sense of freedom, despite the fact that the person had access to staff 24 h a day at the nursing home. The ever-present fear that something might happen restricted the next of kin’s movements. They did not want there to be a drastic worsening of the older person’s condition if they left to go somewhere.

*When I’m on at point of going somewhere, this weekend, for instance, I always think: Oh, just as long as nothing happens while I’m away.* (Daughter)

One consequence of the fact that the older person was always on their mind was that the next of kin found it difficult to delegate any responsibility to other people in the family who were ready to help.

*Even though it’s not up for consideration at the moment, I’ve got a brother at home in case anything should go wrong, but... It’s always on my mind, it’s the way I am. I go to the shops because ‘Mum needs new flowers’ or ‘there’s a summer feast tomorrow, perhaps she should have something nice to wear’. So, she’s never out of my thoughts.* (Daughter)

#### Feeling alone

When a spouse moved to a nursing home, the other spouse could feel lonely because his or her life companion was absent and missed.

*I sit here and doze off in front of the TV, and then, I get angry because he hasn’t woken me up* [laughs]*. I mean, I think he’s sitting there...* (Spouse)

The feeling of being alone was sometimes heightened when there was no other close relative or friend to share the responsibility with. Often, the son or daughter who lived closest to an aging parent had to bear most of the burden. Things became easier, however, after the move to the nursing home, because the next of kin was no longer responsible for the aging parent’s laundry, garden, and home. The tasks that still had to be performed were reviewing the older person’s papers and maintaining the necessary contact with the authorities.

The feeling of being alone could also arise when other members of the family did not involve themselves as the next of kin thought that they should—for example, they did not write in the nursing home’s guestbook. Poor communication among family members often led to frustration regarding the continuity of visits to the nursing home and decisions concerning the older person’s care and welfare. One next of kin spoke of her disappointment with her siblings for not taking responsibility and for not seeing, or not wanting to see, that their mother was “in a prison”. She felt that she was the only family member who went to see their mother, and she felt abandoned by her siblings. One next of kin spoke of meaningless acts by other family members, such as giving the older person a TV, which he or she could not use without help.

*Yes, of course, it’s maybe all right, but I mean, he can’t use the box because it has 20–30 buttons on it. So, he doesn’t get any joy out of the TV any more, and that worries me an awful lot.* (Spouse)

In certain cases, the feeling of being alone was combined with the fact that the next of kin was fully occupied with his or her own life and had children to care for and full-time work but was still the person who was expected to help the older person. The next of kin might then just let the phone go on ringing so as not to have to discuss the older person’s needs or listen to a sibling’s expression of concern about their parent. This was a trying situation.

*The fact that she’s anxious means that I’m more or less assigned the role of the one who gets things done. I try hard not to always take on that role. When I’ve been working all day, after work, I’m a football and indoor bandy trainer for my children. So, in the end, I come home pretty tired. Then, there goes the phone! And it’s my sister who’s worried about something to do with Mum. It’s pretty trying having to go through it all again. So, sometimes, I just don’t answer when she rings.* (Daughter)

#### Couples’ different life situations

The situation where one spouse was dependent on help and the other was not gave rise to pangs of guilt. The one who was independent was faced with having to find the right balance between his or her own well-being and commitment to the partner. After the long effort to meet the extensive care needs of a spouse at home, there comes the realization that the situation has become too difficult to manage, which is followed by the application for admission to a nursing home. All of this is strenuous for both spouses, especially when the dependent spouse wants to go on living at home.

*You feel it. You sit there and there’s not much going on around you, and you think that now’s your chance. Yes, now’s my chance, and I put all of that out of my mind and find something that’s fun to do. We’ve gone on walks, my sister and I, and it’s great, wonderful, but then, there’s always that awareness that I must go there and see how he’s doing.* (Spouse)

The next of kin who were middle-aged spoke of many situations where they felt great responsibility for both of their parents. It might be that one of the parents was in a nursing home while the other parent was dependent on the home help service. This meant that the next of kin had to be in two places, which was burdensome*.* Their leisure time was marked by a fixed routine with much driving back and forth. The parent who lived at home needed shopping to be done and needed company, and the parent who was in a nursing home would expect regular visits.

### Challenges in the relationship

#### Enabling good relations

The next of kin were protective of the older person and sought to ensure that this person’s individuality was respected and allowed to fully express itself.

*You know, it’s about being aware of even the most trivial things that distinguish a human being. It can be whatever shoes are on the person’s feet, for example — this can mean a completely different starting- point for this meeting. It’s about such a little thing as shoes or the colour of a piece of clothing.* (Daughter)

The next of kin saw it as an uncommon privilege to have both parents still alive when they themselves were over 65 years old. If the next of kin was retired, they could go and see their parent at the nursing home at least once a week. It worked out well even if the next of kin was involved in club activities and it was a long drive to the nursing home; it became part of the weekly routine and was no great burden. It was more difficult if the next of kin was still working full-time and had to ask for time off when their parent needed help or otherwise needed to use their ordinary days off. If there was essentially no one else who was close to the older person, the next of kin felt responsible for both the human and the physical closeness.

*Dad’s moved here, from where he grew up, and now, he’s in a nursing home. I’m glad he came here and he’s well looked after, so now I don’t have to go back and forth several times a month to help him with a whole lot of practical things. I’m glad he’s getting help with his practical needs.* (Daughter)

In another case, what had once been a strained relationship became better as time passed, especially after the mother had moved to a nursing home. The daughter realized one day that she was expending too much energy on being angry all the time and that she was no longer up to it.

*So, it just struck me that, no, her brain’s ruined and she doesn’t know any better and that’s the way it is now, and I’ve just got to, well, be a kind daughter to my mother whose brain’s already ruined. I can tell you, I think we’re much, much, much closer to each other now.* (Daughter)

#### Relationship marked by a guilty conscience

The next of kin could also have a guilty conscience without real cause because the older person’s condition had worsened. This was especially the case when the older person behaved badly because of dementia.

*It touched me too closely even though I didn’t want it to. That’s the way I felt, and I’d never felt that way before. But it’s been so overwhelming, and then, of course they know anyway. There’s nothing to be ashamed of, but I’ve felt a bit ashamed anyway. I don’t know why… well, yes, because he’s become so ill.* (Spouse)

Sometimes the next of kin could no longer communicate with the older person who, for example, might be unable to speak or might be living in the past. This could be trying and saddening for the next of kin, which, in turn, gave them a guilty conscience. What was frustrating was not that the person was old but that it was almost impossible to obtain a proper answer regarding how they were feeling. The answer could be in a monotone voice and difficult to interpret. The frustration was worse if it was difficult to reach the staff at the nursing home who could provide information concerning how the person was getting on.

*What’s trying isn’t that Mum’s ageing. What’s trying is that she can’t carry on a conversation. Most of the time, when I ask her something, she says ‘Right’. That’s all she can say: ‘Right’. It can sometimes be a bit difficult to ask anything on the weekends when there are so few staff. There should be more staff.* (Daughter)

The next of kin who were themselves old and whose health was failing were disappointed and sad that they did not have the energy to go to the nursing home as often as they wanted. The guilty conscience was also very noticeable in the case of the next of kin who had a full programme of leisure activities and whose aim was to neglect nothing in their everyday lives, but they still needed time to go to the nursing home. The next of kin spoke of the difficulty of being emotionally placid in the encounter with the older person, especially if the older person was living in a past to which the next of kin had no access.

*Yes, that’s it. You can sometimes have a guilty conscience because you did not get enough sorted out. Sometimes it’s pretty trying going there because of course she’s old and I’ve heard her stories over and over and over again and, in some way, she’s back in the old days and talks about her dad and all that.* (Daughter)

#### Encountering vulnerability

When the older person became physically worse and the future looked uncertain, the next of kin felt deeply frustrated. There was a constant sense of one’s own inadequacy, considering that the older person and their own children and grandchildren, who were in the prime of life, all needed support.

*When Dad isn’t all right, I’m not all right either. He’s much better now than he was a month or so ago, so that makes things a bit calmer anyway. At that time, I went to see him every day and you didn’t really know what was going to happen* [laughs a little]. (Daughter)

The next of kin described difficulties in handling the older person’s vulnerability caused by mental deficiencies. It was worrying and emotionally disturbing when the older person withdrew into himself or herself and did not have the energy for proper contact. There was a feeling of helplessness in the face of the older person, with a loss of interest in life.

*But Mum’s the sort that, when she’s in low spirits, she just wants to go to sleep and not wake up again, and that can be hard to cope with. She’s always been close to my sister, but now they’re not in touch any more.* (Daughter)

Focusing on tasks that had to be done was a way of coping with the fact that the older person was no longer the same as he or she had been before. The following statement illustrates how trying it is to be in a state of readiness to help the older person 24 h a day.

*I go and see my father on Wednesdays and Saturdays, and then, he tells me what he wants me to get for him. Now, he’s started ringing during the night because he can’t see what time it is. He gets very confused, doesn’t know whether it’s morning, noon or night. It’s been like that for 5 years, which is to say, ever since he came here.* (Daughter)

#### Strained relations

The nature of the past relationship between the older person and the next of kin played a part in determining the present relationship. A previously strained relationship became even more strained if the next of kin was, for example, working full-time or ill. In the case of earlier strained relationships, the next of kin wondered about their own part in this tension. Although the next of kin understood the older person’s difficulties because of severe pain, this was not seen as excusing the older person.

*Just because she’s old, she can’t behave any way she wants. I don’t think it gives her the right to have outbursts of anger towards family.* (Daughter)

A daughter found it upsetting and frustrating that her mother did not take care of herself as she should. The daughter could not get her mother to eat the right type of food, much less get her to make the changes in lifestyle that are required to manage diabetes. The mother had turned “a deaf ear” to the dietary advice offered both by the daughter and the staff. This was saddening and had a negative effect on the relationship between the mother and daughter. Other next of kin coped with a bad relationship by defending their own priorities in everyday life.

*Feelings have come to the surface now that have been in existence for a long, long time. Yes, and then I think, well, when he’d turned 60, I say to myself: ‘I’ve got quite enough on my plate with my own children and grandchildren. I can’t use up all my energy on the older generation’. Yes, that’s a bit how it feels.* (Daughter)

### The significance of the quality of care in a nursing home

#### Satisfaction and appreciation

It was appreciated by the next of kin that the care was of a high quality, which made their lives easier. It was felt that the staff were *there* for the older person and did all that they could. It was a great relief to be able to share the burden, communicate with the staff and collaborate with them in seeing that the older person’s needs were met. It was also much appreciated when the staff knew about the person’s past life and could relate to it. This reinforced the next of kin’s perception of the staff as being able to offer high-quality care. Especially satisfied were the next of kin who lived near the nursing home and thus had no trouble getting there or fetching the older person for a visit.

*Yes, it gives me a sense of security. Because it’s hard enough anyway, being a mother to your own mother.* (Daughter)

#### Lack of person-centredness

Although the next of kin perceived nursing home staff as being capable, there was a certain anxiety and resignation concerning the fact that the staff did not have the opportunity to provide “that little extra”; for example, they did not have the time to stop and chat with the older person or read the paper to them. The next of kin were concerned as to whether the staff really valued the older person as a unique individual—for example, whether the staff appreciated that the person made great efforts to be clean and to have nice clothes to wear. There was disappointment that the Life History (*Levnadsberättelsen*) (which is composed by the next of kin in conjunction with the move to a nursing home and indicates the older person’s preferences) had hardly ever been used. It was saddening and frustrating that the staff, with certain exceptions, had not used the information and were incapable of seeing the older person as the person he or she was.

*It’s so important that they’ve got nice shoes or something. It doesn’t take much to raise a person’s spirits.* (Daughter)

Since the majority of the residents were too ill to carry on a conversation, life at the nursing home could become tedious for the people who were healthier and eager to talk. Furthermore, the goals that had been established in conjunction with the care plan were not always respected by the staff, which meant that the older person’s needs were not fully met. The next of kin expressed fear that even older persons with undiminished cognitive capacity were at risk of being rendered passive.

*Yes, of course, I’ve seen tendencies in the people who come here, who’ve been pretty hale and hearty but become more and more withdrawn, become silent; it’s very hushed all around. I can tell you, though, that the staff are good, so I must say it’s been better here than I’d expected.* (Son)

The staff did not have time, or did not take the time, to go out with the older person or arrange exercises designed to make it easier to cope. Instead, they offered assistive devices such as a rollator (a walking frame equipped with wheels). The next of kin felt that the older person did not receive natural training regarding using their body in everyday life and that the staff were more interested in their own ergonomics. Clearly, the next of kin thought that the older person was being rendered passive by not being allowed to do anything or take any responsibility. There was a sort of humdrum routine where the older person did not have to think about anything whatsoever.

*She doesn’t have to take responsibility for anything, so she doesn’t have to take responsibility for going to bed, she doesn’t have to take responsibility for getting up, she can just exist, so to speak… Before, she had to go and cook coffee for herself, and stuff like that.* (Daughter)

The next of kin sometimes felt a great sense of responsibility with regard to making use of their own experience and understanding to enhance the older person’s care, but it was difficult to find a balance so that the staff did not see them as bossy and too demanding. They felt great frustration with the staff’s restricted opportunities to become familiar with the older person’s needs. They finally gave up on the idea of improving the older person’s situation and focused instead on making their visits to the nursing home *special occasions*. There were situations where it was difficult to do one’s best for the older person.

*Well, I mean, I can’t influence everything. I mean, I’m not there. I live a long way away.* (Daughter)

The staff were not always obliging when it came to help with practical matters, such as turning on the older person’s TV. It was particularly depressing when the older person wanted to watch a favourite programme and the staff would not take the time to turn it on but instead made a big show of being busy with other things. It was distressing when the care was insufficient and ineffective when it came to basic needs such as receiving help in the case of physical weakness or diminished vision.

*Well, we’ve been anxious and worried about her even though she’s been pretty self-sufficient before. Then, suddenly she’s much, much, much weaker, and of course, we’ve had a few things to say before about their not taking into proper consideration how disabled she is by having such poor sight.* (Son)

The next of kin spoke about the responsibility for care in connection with the worsening of the older person’s state of health. It was felt that the nursing home staff lacked the ability to perceive that a situation had become acute and to act on this perception. It was also felt that the decline in the older person’s well-being was not given a high priority.

*I’ll be coming with her again tomorrow, of course, because she can’t fix this. I know my mum. I was allowed to take her back to the nursing home, but then she just lay there. That’s how it was, and she just got worse and worse; so yesterday, we had to go to the Emergency again.* (Daughter)

It was also a torment for the next of kin to observe that the older person did not receive proper palliative care in life’s final phase.

*I know she’s old and ill in every way, but she still has a right to receive proper care at the end.* (Son)

## Discussion

The study explores QoL among the next of kin of older persons in nursing homes before the implementation of knowledge-based palliative care. The main finding when it comes to experienced QoL shows positive and negative feelings with regard to being the next of kin of an older person. The next of kin experienced a transition due to the new life situation caused by the older person’s health condition and care in a nursing home and the related changes for themselves. Despite the next of kin’s rich narrations concerning the care for their frail older family member, they did not use the term palliative care in their narrations, and very few of them used the words dying or death. Their QoL was also related to the previous and current relationship with the older person living in the nursing home and the quality of the care that was offered there.

Our findings illustrate several aspects of the orientation to the new life situation that could either increase or decrease the next of kin’s QoL. The next of kin experienced a feeling of relief when the older person went into a nursing home and had a sense of security in knowing that there were staff available 24 h a day. At the same time, however they found it difficult to stop thinking about their loved-one’s dependency on the staff in respect of the quality of care, which induced anxiety. The experiences and efforts to cope with the new situation observed in this study have been described before in studies on the transition into a nursing home. When the older person’s increased need for care has led to care at a nursing home, this event can be perceived as a crisis process for family members, with a turning point, a coping phase, and an outcome of the crisis process [29] The situation is ameliorated if the next of kin are regarded as partners in care and if regular meetings with staff are offered [29]. A Norwegian study [1] found that the next of kin’s attempts to cope with this new situation often considered previous experiences and were characterized by uncertainty, expectations, and mixed feelings of relief and concerns about the quality of care. The participants also stated that they still felt responsible and continued to perform the caring tasks that they had performed when the older person lived at home. A Swedish study of the next of kin of older persons who have died at a nursing home [5] describes that the nursing home placement meant that the next of kin were no longer the main guardian, which resulted in security and less worry for them.

Another aspect of the adaptation to the new life situation in this study was that the next of kin felt alone, which was evident in spouses who had previously lived with the older person. The feelings of being alone were heightened when there were no other next of kin or if there were other next of kin but they did not share the responsibility. Loneliness is a common problem among older persons [30]. In a Swedish study [31] of 4278 persons aged 75 years, loneliness was the most important factor that predicted a low QoL among both non-caregivers and older people in general. This fact implies that staff need to pay special attention to older next of kin who have previously resided with the older person when that person moves to a nursing home. Healthcare professionals need to understand that next of kin who experience loneliness are at a greater risk of developing both physical and psychological ill health and that it may adversely affect their QoL [30, 32–34].

The findings relevant to the theme “challenges in the relationship” in this study illustrate that the quality of the relationship in the past affected the possibility of creating a good relationship in the present. Changes in the older person’s health status could also change the prerequisites for continuing a relationship with the older person as before. The progression of a diagnosis, such as dementia, could jeopardize a relationship due to the difficulty of communicating with one another about important things such as the older person’s wellbeing, needs and wishes, as seen in our study. The next of kin attempt to continue the relationship with their nursing home resident because of its importance and for the purpose of “making it better” [31, 32]. According to Garity [35] other forms of interaction could be useful in these situations to maintain the relationship in some way, such as singing together. The study of Strang et al. [36] describes that being the only relative who is recognized by the older person as the next of kin could increase the pressure to visit the nursing home, which could affect the relationship in a negative way.

The next of kin in our study described having a guilty conscience, which is corroborated by Graneheim, Johansson and Lindgren [29] as feelings of guilt and shame for no longer being able to be the informal caregiver at home. The authors relate these feelings about the decisions that must be made to change the care venue of the older person to feelings regarding the change in the next of kin’s own role as informal caregiver. The consequences of the decision were described as a process for both the next of kin and the older person. Graneheim et al. [29] claimed that if the next of kin were involved in care and became partners with the staff, then this would have a positive effect on the next of kin’s QoL.

The next of kin encountered vulnerability in terms of the older person’s impaired health as painful and frustrating, which increased their worry and need to monitor the care because they wished for their family member to receive dignified care. The staff’s availability and good communication with the next of kin were important. The next of kin feared neglect of the resident’s needs when they left the nursing home and did not know what was expected from them as family members; thus, making decisions was not easy. In a review study by Fosse, Schaufel, Ruths and Malterud [37], similar experiences were described: the next of kin felt relieved when the staff recognized the older person’s needs and offered the necessary help. Grøn [38] indicates that an older person’s vulnerability is complex to understand. How this vulnerability is interpreted and perceived depends on the perceptions of the older person, the next of kin and the staff. One can be regarded as a vulnerable person by other people (from an outside perspective) but not feel vulnerable oneself, despite severe illness and old age. Other studies confirm that the concept of vulnerability is not well defined and understood but that staff should be given knowledge regarding how to offer more holistic care and, in this way, prevent vulnerability in care [39, 40].

In our results, it was difficult for the next of kin to deal with the older person’s declining health status. Hvalvik and Reierson [41] also described that the next of kin can experience difficulty accepting such changes, and it is important that these feelings are taken seriously by the staff. Feelings of uncertainty and insecurity could cause emotional stress and frustration together with feelings of an overall responsibility for care. Being ready to act could be interpreted as balancing vulnerability while simultaneously managing challenges that make the next of kin feel strong and proud [41]. From an ethical perspective, Morberg and Jämterud [42] argue that care can be dignified by sharing human vulnerability through presence, responsibility and answerability.

In addition, the QoL of the next of kin was directly dependent on the quality of care that they perceived the older person was offered. Other studies have confirmed the relation between the QoL of the patient and that of the next of kin [43, 44], noting that the quality of care, with a good relationship among the staff, next of kin and the ill person, was important [41]. In our study, it was expressed that when everything worked well, it gave the next of kin a sense of security; however, when the care was not provided as expected, it resulted in anxiety. This was evident when the care did not originate from person-centred care and the life story of the older person. Lavoie, Blondeau and Martineau [45] found that a changed focus from tasks that need to be performed in care to more person-centred care shifted the priority to the choices, desires and needs of the ill person. Person-centred care even affects the relationship and communication between the staff and the next of kin in a positive way. Similar to the findings in our study, the next of kin of nursing home residents in the UK [46] reported that “the little things” were important for the quality of care and that if they were neglected, it was a source of distress. The next of kin in our study expressed that they wanted to participate in the care, but it was difficult to find a balance where the staff did not see them as demanding. Other studies that describe the next of kin’s situation in nursing homes and in EoL care [6, 10, 46] have shown that the next of kin need to supervise the care to ensure that it is correctly performed. However, it can be difficult for the next of kin to maintain control, especially if they do not share suggestions about care because of fear that their supervision could upset staff [47]. In our study, visiting the nursing home enabled the next of kin to monitor the care and support residents in circumstances that threatened their dignity.

The interviews with 40 next of kin who live in two separate counties (20 from each county) were conducted before the implementation of knowledge-based palliative care [22] and contribute to knowledge about the next of kin’s experience of the QoL at baseline. The participants in this study were chosen to explore a diversity of experiences, which increases the credibility of our results [48, 49]. Interviews were conducted with next of kin from both large and small nursing homes situated in both urban and rural areas. Therefore, the results are transferable to a broad population.

The interviews were conducted by four different researchers, which may have had a negative effect on the credibility of the findings. However, the researchers used the same interview guide and had regular meetings with the experienced project manager (GA) for one to 2 h each week to discuss different aspects of the data collection, including the interview process. Furthermore, the interviewers were all nurses with considerable experience in elder care. The two researchers who conducted the analysis did not perform the interviews, which could be seen as both a strength and a weakness of the study. They interpreted the text independently of one another and had several meetings together, i.e., investigator triangulation. After the preliminary analysis, two of the researchers who conducted the interviews confirmed the analysis. The analytical process was carefully described to enable the reader to follow the researchers’ interpretations, which strengthens the credibility of the study. Another method that increased the credibility was the use of quotations. The quotations were selected from a variety of next of kin with different relationships to the older per.

## Conclusions

The study explores QoL among the next of kin of older persons in nursing homes before the implementation of knowledge-based palliative care. The findings show that the nursing home can be perceived as an enabling context for good relations. Commonly, the QoL experiences consisted of feelings of burden due to the quality of the relationship with the older person and concern about the older person’s deteriorating health and low quality of care. The next of kin were aware of the older person’s worsening health condition but did not use words such as palliative care or dying. These findings indicate the importance of increased knowledge of and training in palliative care in nursing homes to better satisfy the next of kin’s needs.

## Data Availability

The Regional Ethics Review Board in Lund established limitations regarding the accessibility of the data in the KUPA-project. The data generated and analysed in this sub-study of the KUPA-project are therefore not publicly available due to the inclusion of sensitive information. Before approving access to the data, the principal researcher (GA) of the KUPA project must consult with the ethical review board.

## Abbreviations

EoL: End of Life
EoLC: End of Life Care
KUPA: An acronym from Swedish: KUnskapsbaserad PAlliativ vård
QoL: Quality of Life
WHO: World Health Organization
